# The Role of Eosinophils in Bullous Pemphigoid: A Developing Model of Eosinophil Pathogenicity in Mucocutaneous Disease

**DOI:** 10.3389/fmed.2018.00201

**Published:** 2018-07-10

**Authors:** Kyle T. Amber, Manuel Valdebran, Khalaf Kridin, Sergei A. Grando

**Affiliations:** ^1^Department of Dermatology, University of California, Irvine, Irvine, CA, United States; ^2^Department of Dermatology, Rambam Healthcare Campus, Haifa, Israel; ^3^Departments of Dermatology and Biological Chemistry, Institute for Immunology, University of California, Irvine, Irvine, CA, United States

**Keywords:** bullous pemphigoid, eosinophils, eosinophilia, major basic protein, eosinophil cationic protein, pruritus, cytokines

## Abstract

Bullous pemphigoid (BP) is an autoimmune blistering disease which carries a significant mortality and morbidity. While historically BP has been characterized as an IgG driven disease mediated by anti-BP180 and BP230 IgG autoantibodies, developments in recent years have further elucidated the role of eosinophils and IgE autoantibodies. In fact, eosinophil infiltration and eosinophilic spongiosis are prominent features in BP. Several observations support a pathogenic role of eosinophils in BP: IL-5, eotaxin, and eosinophil-colony stimulating factor are present in blister fluid; eosinophils line the dermo-epidermal junction (DEJ) in the presence of BP serum, metalloprotease-9 is released by eosinophils at the site of blisters; eosinophil degranulation proteins are found on the affected basement membrane zone as well as in serum corresponding with clinical disease; eosinophil extracellular DNA traps directed against the basement membrane zone are present, IL-5 activated eosinophils cause separation of the DEJ in the presence of BP serum; and eosinophils are the necessary cell required to drive anti-BP180 IgE mediated skin blistering. Still, it is likely that eosinophils contribute to the pathogenesis of BP in numerous other ways that have yet to be explored based on the known biology of eosinophils. We herein will review the role of eosinophils in BP and provide a framework for understanding eosinophil pathogenic mechanisms in mucocutaneous disease.

## Introduction to bullous pemphigoid

### Clinical presentation of bullous pemphigoid

Bullous pemphigoid (BP) is the most common autoimmune blistering disease with an estimated annual incidence between 2 and 22 new cases per million people (1–8). BP mainly affects the elderly with an age of onset in the late 70s (8–10). Association with neurological disorders such as dementia, Parkinson's disease and cerebrovascular disease is seen in between 28–56% of BP patients (8, 11).

Most commonly, BP presents with chronic and recurrent blisters, usually arising on urticarial or eczematized skin, favoring the abdomen, and flexural aspects of the extremities. Blisters turn into erosions by mechanical friction with subsequent crust formation and healing. Prior to the development of the blisters, a prodrome of pruritus with or without urticarial lesions commonly occurs (12). Oral involvement is seen in 10–20% of BP patients (13, 14). Pruritus alone may be the only symptom of BP in some of the cases, though it is controversial whether these patients represent falsely seropositive elderly patients with other causes of pruritus, or are in fact pre-clinical cases of BP (15, 16).

Numerous clinical variants exist, with atypical clinical variants accounting for approximately 20% of cases (8, 10, 13, 16). Likewise, medications can also induce bullous pemphigoid, (8, 17–20) with a more atypical clinical and immunologic phenotype seen particularly in patients with dipeptidyl-4 inhibitor induced BP, who demonstrated a decrease in peripheral eosinophil infiltration (21–24).

### Diagnosis of bullous pemphigoid

**Figure 1:**
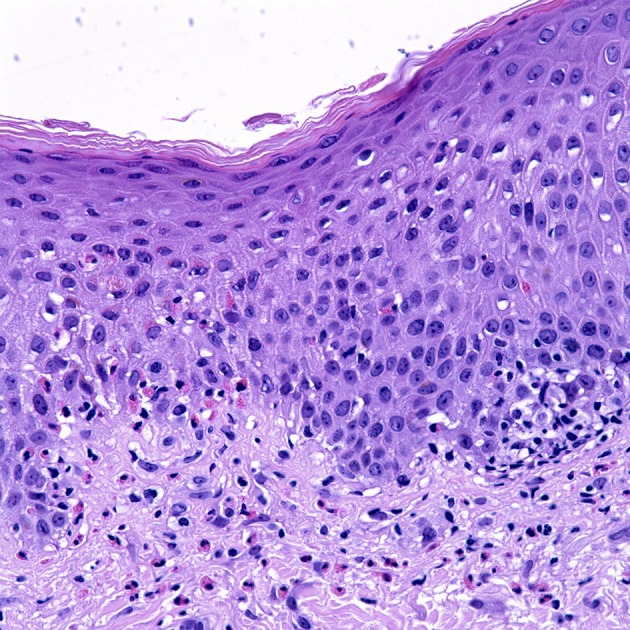
Uriticarial bullous pemphigoid. Histological section shows dermal inflammatory cells, predominantly composed of eosinophils, which line up at the dermoepidermal junction. Notice the spongiosis and exocytosis of eosinophils through the basement membrane into the spinous layer. H&E × 4000. (Courtesy of Dr. Philip LeBoit and the Dermatopathology Service at UCSF).

Histological sections of BP typically show a subepidermal blister with variable degree of inflammatory infiltrate composed of lymphocytes, neutrophils, and characteristically eosinophils. Histological presentation may vary depending on the clinical presentation. Urticarial lesions may present with spongiosis and eosinophils infiltrating the epidermis, also termed eosinophilic spongiosis, with an absence of subepidermal clefting (Figure 1) (25, 26). Peripheral eosinophilia is present in around 50% of treated patients (27–29).

The diagnosis of BP requires further immunology workup in the form, of immunofluorescence and serologic studies. Direct immunofluorescence reveals linear deposition of IgG and complement component 3 (C3) at the dermal-epidermal junction (DEJ); linear IgA or IgE positivity is sometimes appreciated (8, 30, 31). Indirect immunofluorescence (IIF) shows linear deposition of IgG at the basement membrane on monkey esophagus or the epidermal side of salt-split human skin (32). Circulating antibodies against the proteins BP180 and BP230 can be detected in serum samples by ELISA, with sensitivities ranging from 66 to 100% (33–36). Sensitivity of the BP180 non-collagenous 16A (NC16A) domain ELISA is comparable with that of IIF with salt-split skin (37).

### Pathogenesis of bullous pemphigoid

Evidence points to formation of autoantibodies against the hemidesmosomal proteins BP180 and BP230 as the leading events in blister formation in BP both clinically and experimentally (38, 39). BP180 (type XVII collagen) is a transmembrane glycoprotein with an extracellular C-terminus that mediates adhesion between the epidermis and the basement membrane (28). Association of disease activity has been demonstrated to clinically correlate with serum concentration of IgG antibodies against NC16A which is considered to contain the main pathogenic epitope of BP (40). Experiments with cultured human keratinocytes have shown cell detachment with reduced BP180 expression after tissue was incubated with antibodies against BP180 protein (41); moreover, anti-BP180 IgG and IgE induce signal transduction events with upregulation of interleukin-6 and interleukin-8 confirmed at the protein and mRNA levels (42–44). These results have been reproduced by transgenic mice expressing human BP180 (45).

Utilizing this humanized BP180 model, investigators demonstrated the role of T-cells as helpers of B-cells to differentiate into plasma cells and produce autoreactive IgG. Particularly they showed that NC16A-reactive CD4^+^ T cells in these mouse model could activate B-cells to produce anti-NC16A IgG via CD40-CD40L interaction (46). In humans, there is an association between BP and the HLA-DQB1^*^03:01 allele, as stimulation with BP180 to both healthy and BP patients with this allele can exert a T-cell response. Patients with BP, however, demonstrate a Th2 response while healthy HLA matched controls demonstrate a Th1 response (47–49).

BP230 is an intracellular plakin-like protein of the hemidesmosomal plaque (28). The pathogenic role of anti-BP230 antibody has not been found to be as conclusive as anti-BP180 antibody because blisters have not been observed consistently in animal models in which antibodies to BP230 are present. Furthermore, relationship between serum anti-BP230 autoantibody and disease activity has not been clearly established (12). In clinical practice, the use of ELISA for antibody to BP230 protein increases the sensitivity only 5–10% when combined with that for BP180 protein (50–52). Still, anti-BP230-type BP has been described a distinct entity, thus suggesting a pathogenic role of these autoantibodies as well as a potential for synergy with other pathogenic anti-basement membrane zone (BMZ) autoantibodies (53).

Despite the clear pathogenic role of anti-BP180 IgG autoantibody, many clinical manifestations and pathways are not easily explained by this alone. Effector cells of cell-mediated autoimmunity must be considered as significant contributors to the pathogenesis of BP. Eosinophils, have several known pathogenic roles in BP. Likewise, several known functions of eosinophils that have yet to be described in BP may play an additional role in the pathogenesis and symptoms of BP. We will review both known contributions of eosinophils to the pathogenesis of BP, as well as known mechanisms of pathogenic action of eosinophils that have yet to be evaluated specifically in BP.

## Introduction to eosinophils

Eosinophils are effector cells found in various organs including the skin. Their impact on biological processes is likely mediated primarily by their cytoplasmic granules. These granules are classified as primary, secondary, small granules, and lipid bodies (54). Secondary granules contain four toxic basic proteins: the major basic protein (MBP), eosinophil peroxidase (EPX), eosinophil derived neurotoxin (EDN) and eosinophil cationic protein (ECP). The crystalloid core of secondary granules is constituted by highly cationic MBP and is covered by the 2 ribonucleases: ECP and EDN (55). ECP is used extensively as a marker to assess activity in various inflammatory diseases (56). Granules are secreted when eosinophils become activated. These granules are highly toxic to microbes, parasites, and tumor cells (57). For instance, ECP is a cytotoxic ribonuclease with the ability to exterminate parasites, bacteria and virus *in vitro* (56). Moreover, ECP forms pores or transmembrane channels, which ultimately results in cellular damage and death (58). ECP can also lead to epithelial and neuronal apoptosis (59–61). MBP toxicity is mediated by affecting the charge of cellular surface membranes resulting in disruption and altered permeability leading to cellular injury (54, 62). Eosinophils are also implicated in the production of various cytokines, chemokines, lipid mediators, and superoxide. Complex immunomodulatory functions have been attributed to eosinophils which can also act as antigen-presenting cells (APCs) (57).

Peripheral eosinophilia is the result of the secretion of several factors such as IL-5, granulocyte-macrophage colony-stimulating factor (GM-CSF), and IL-3 (63). Migration of eosinophils from circulation into the skin is mediated at least in part by very late activation antigen-4 (VLA4) which is expressed in eosinophils and binds to vascular cell adhesion molecule 1 (VCAM-1) on vascular endothelium. Other chemoattractants include eotaxin-1 (CCL11), eotaxin-2 (CCL24), eotaxin-3 (CCL26), RANTES (CCL5), and monocyte chemoattractant proteins which can bind to eosinophils and lead them to lesional sites (57, 64, 65).

### Known mechanisms by which eosinophils can contribute to the pathogenesis of bullous pemphigoid

There are several lines of evidence suggesting the role of eosinophils in the pathogenesis of BP. Peripheral blood eosinophilia is present in ~50% of affected patients (27–29). Furthermore, elevated serum concentrations of secretory granules, such as ECP, are significantly elevated in patients with BP, with levels paralleling disease severity (55, 66–71). A similar relationship has been documented to occur with IL-5 levels which runs parallel not only to disease severity, but also to ECP levels (68, 72–80). This is consistent with an increase in eosinophil activation, as confirmed by expression CD69, in peripheral blood and lesional skin of BP patients (81). While an increase in blood and tissue eosinophils has long been known (82), the actual role of eosinophils in the pathogenesis of BP is becoming more readily understood.

### Production of metalloproteases

Proteases including gelatinase B (92-kD gelatinase, matrix metalloproteinase [MMP]-9) and neutrophil elastase (NE) play a significant role in degrading BP180 and cleaving the DEJ (83). MMP-9 is released as a zymogen and is subsequently activated by a series of proteases including MMP-2, 3, 7, 10, and 13, as well as cathepsin G, plasmin, and trypsin. MMP-9 intervenes in tissue remodeling and facilitates cellular migration, extracellular matrix degradation and tissue destruction (84). Studies in isolated human eosinophils have documented that tumor necrosis factor-α (TNF-α) is a potent stimulator for a rapid release of pro-MMP-9 (85). Experiments in peripheral blood of allergic volunteers demonstrated that IL-3 in combination with TNF-α induces significant MMP-9 synthesis by eosinophils (84).

MMP-9 can cleave the extracellular collagenous domain of recombinant 180-kD BP antigen (86). Eosinophils appear to be the principal culprit in MMP-9 secretion. Strong signal for gelatinase mRNA has been detected in eosinophils but not in neutrophils at site of blister formation. *In vitro* studies conducted in Matrigel have likewise demonstrated eosinophils' ability to degrade the BMZ, identifying MMP-9 as the key protease. Notably, release of MMP-9 was increased only in the presence of both IL-5 and platelet activating factor (PAF) (87).

The direct role of MMP-9 in cleaving BP180 has been challenged, based on its ability to regulate neutrophil elastase (NE). In mouse models, MMP-9 regulates NE activity by inactivating α1-proteinase inhibitor, thus contributing to further degradation of BP180 and DEJ separation (88). Studies by Verraes et al showed that despite the presence of the proform of MMP-9 in human lesional skin, BP180 degradation could be inhibited by a specific elastase inhibitor, but not by a wide spectrum of matrix metalloproteinase inhibitor, suggesting the importance of the regulatory role of MMP-9 on NE in blister formation (83).

### Production of eosinophil degranulation proteins

Eosinophils and neutrophil granule proteins can be detected in the blister fluid and serum of BP patients (55, 66, 67, 89). Moreover, studies have found peroxidase positive eosinophil granules along lamina lucida of the BMZ in BP patients (90). Scanning electron microscopy studies have revealed granule release into basal cells (91). Eosinophil granule protein deposition has been demonstrated not only in fully developed blisters, but also at the earliest stages of blister development and urticarial lesions of BP (73, 92). Eosinophil degranulation has also been observed in pemphigoid gestationis, a gestational variant of BP, whereby MBP is deposited extracellularly in the dermis (93).

More recent *in vivo* observations have demonstrated the presence of ECP, EDN, EPX, and MBP in skin. In addition, *in vivo* experiments in guinea pig skin have demonstrated the presence of these granules for weeks after intradermal injection at concentrations seen in human disease. During these experiments, Davis et al demonstrated increased cutaneous vascular permeability as an effect of the degranulation; however, basement membrane and epidermal alterations such as spongiosis were not examined (94).

*In vitro* experiments have shown that eosinophils may be activated through augmenting cell-surface receptors and receptor-linked oxidative metabolism (95). Upon activation, eosinophils degranulate (55, 66, 67, 89). Tsuda et al. demonstrated that degranulated eosinophils adhered to basal keratinocytes suggesting that eosinophil granules may directly damage basal keratinocytes leading to DEJ separation (89). We have demonstrated that MBP has a concentration dependent cytotoxic effect on cultured keratinocytes (96).

As eosinophils also release tissue factor (TF), an initiator of blood coagulation, Marzano et al, hypothesized a role for local activation of the coagulation cascade in BP. This was confirmed to be the case (97), with subsequent correlation between ECP levels and prothrombotic markers (70). Cases with higher coagulation, like ECP, are associated with more severe disease (97). Whether this contributes to blister development is, however, unclear.

### Production of eosinophil extracellular traps

Relevance of extracellular traps produced by eosinophils (EET) have increased, at least in part due to the interesting findings obtained from studies on neutrophils. Extracellular traps consist of network-like structures containing DNA, granule proteins and nuclear proteins. These traps can expand up to 15 times the size of the cell, thereby increasing the effective targeting area (98). Experiments performed on skin biopsies from human participants have shown that EET are present in infectious skin diseases, allergic diseases and autoimmune diseases including BP. The number of eosinophils releasing DNA appear to be around 10%, though this phenomenon was most commonly observed in Well's syndrome, whereby trap formation was seen in up 30% (99). *Ex vivo* experiments utilizing human skin and isolated human eosinophils have shown that EET may contribute to DEJ splitting, after the observation that DNase significantly reduced DEJ separation (99). Still, the mechanism by which EET contribute to DEJ separation is unclear.

### Link between anti-BP180 IgE autoantibodies and dermo-epidermal junction separation

Evidence supporting the pathogenic role of IgE autoantibodies in BP as well as its relationship with eosinophils has increased in recent years. Passive transfer of anti-BMZ IgE autoantibodies results in erythema, pruritis, eosinophil infiltration, and histologic blistering (100). This study did, however, use the LABD97 portion of the BP180 protein rather than the NC16A portion. Lin et al. created a transgenic mouse model expressing human hNC16A and human high-affinity IgE receptor (FcεRI), showing that anti-NC16A IgE from BP patients induced subepidermal split as well as eosinophil infiltration and IgE deposition at the DEJ. Particularly, they found that eosinophils are essential in order to induce BMZ separation in the presence of anti-NC16A IgE *in vivo*. This step appears to be key in DEJ separation, thus supporting the pathogenic role of IgE autoantibodies against the NC16A region of the BP180. Likewise, eosinophils in this animal model expressed FcεRI, thus providing a further link between IgE autoantibodies, eosinophils and blister formation, occurring independently of neutrophils (101).

The expression of FcεRI in monocytes, mast cells, basophils, eosinophils, dendritic cells, and platelets has been highlighted recently as a link between the biology of these cells in the presence of IgE autoantibodies (102). FcεRI, the high affinity IgE receptor is typically minimally expressed on eosinophils, but it is highly expressed on eosinophils in BP and other diseases characterized by high IgE and eosinophilia (103). FcεRI consist of an α-chain, which controls IgE binding, and β- and γ-chains, which intervene in signal transduction. These chains have been found in either tetrameric (α*βγ*_2_) or trimeric (αγ_2_) form in humans (103). Interestingly, investigators have found mRNA for α-,β-, and γ- chains in eosinophils of BP patients. These FceRI eosinophils appear to predominate in the dermis rather than epidermis (104). The capability of eosinophils to bind IgE may thus influence their subsequent degranulation (103).

Elevated serum IgE in patients with BP was first described in 1974 (105). Since then, several studies have demonstrated elevated IgE levels in up to 40–50% of patients, with the notable discovery of anti-basement-membrane IgE autoantibodies (29, 106–108). Advancements in technology led scientists in the 1990s to identify IgE autoantibodies to different BP molecules such as BP230 (109, 110). Later in the 2000s, IgE autoantibodies targeting NC16A, the principle epitope of BP180 to which IgG4 antibodies preferentially react, were described (111). IgE reactivity with other epitopes, particularly the intracellular domain of BP180 was subsequently described (112). This intracellular domain is critical for incorporating proteins into hemidesmosomes, raising the possibility that autoantibodies against it could impair the interaction of BP180 with other constituents of the hemidesmosome (112). Experiments conducted by Freire et al provided evidence that IgE and BP180 form immune complexes in BP skin. Furthermore, *in vitro* experiments found that IgE-BP180 complexes have the potential to cross-link FcεRI in basophils leading to activation and degranulation (113). Approximately half of patients demonstrate IgE autoantibodies against BP180 and BP230 (114). Ultimately, the use of an eosinophil deficient mouse demonstrated that eosinophils are the essential link between anti-BP180 IgE antibodies and BP (101).

Despite the prevalence and clear causal role of anti-BP180 IgE in inducing BP, the clinical significance of these autoantibodies remains unclear (114). A systematic review of studies associating disease phenotype with the presence of anti-BMZ IgE found no association with phenotype, though the presence of anti-BMZ IgE was associated with greater disease severity (115). Treatment results with omalizumab, a monoclonal antibody blocking soluble IgE from binding to its receptors, have been mixed with moderate efficacy, showing only a limited role as a monotherapy (116–118). As anti-BP180 IgE ELISA is not routinely available, selecting BP patients for this treatment regimen remains a challenge in the routine clinical practice.

### Production of key cytokines and chemokines

Evidence of both inflammatory and anti-inflammatory cytokines have been described to be present in BP. Elevated levels of proinflammatory (IL-6, TNF-alpha, IL-8) and anti-inflammatory cytokines (IL-4, IL-10) have been reported (119). Moreover, Giomi et al has suggested that cytokine milieu varies according to the chronicity of BP. An initial Th0/Th2-like response would be seen in early stage of BP with IL-4, IL-5, and low levels of IFN- γ. Whereas in chronic phases, a Th1 response would follow with significant expression of IFN- γ (120).

A complex network of chemokines also contributes to the development of BP. Both Th1 and Th2 chemokine profiles are exhibited in BP as follows: macrophage inflammatory protein-1 β (MIP-1β) and IFN- γ- inducible protein 10 (IP-10) for Th1; and eotaxin, monocyte chemoattractant protein (MCP)-4 for Th2. Overall, there is predominance of Th2 chemotactic activity compared to Th1 cells (79). Other studies have shown significantly levels of MCP-1, and IP-10, monokine induced by IFN- γ (MIG) for Th1 (121), and, eotaxin, CCR3, and MCP-4 for Th2 (77, 79, 122). Experiments conducted by Gounni-Abdelilah et al found that eotaxin and MCP-4 were present in eosinophil granules in the bullae of patients with BP, as well as in the epidermis and infiltrating eosinophils in skin of BP patients and these were secreted by eosinophils when stimulated by IgG, IgA, or IgE immunocomplexes (79). This autocrine pathway may thus perpetuate the immune response in BP, leading to chronicity of lesions (79). Cytokines and chemokines known to be produced by eosinophils are summarized in Table 1.

**Table 1:** Cytokines and chemokines capable of being secreted by eosinophils (65).

**Cytokines**	**Chemokines**
A proliferation-inducing ligand (APRIL)	CCL3/macrophage inflammatory protein-1α (MIP-1α)
Granulocyte/macrophage colony-stimulating factor (GM-CSF)	CCL5/RANTES
Interleukin-1α	CCL11/eotaxin
Interleukin-1β	CCL13/monocyte chemoattractant protein-4 (MCP-4)
Interleukin-2	CCL17/thymus activation regulated chemokine (TARC)
Interleukin-3	CCL22/macrophage-derived chemokine (MDC)
Interleukin-4	CCL23/myeloid progenitor inhibitory factor 1 (MPIF-1)
Interleukin-5	CXCL1/Groα
Interleukin-6	CXCL5/epithelial-derived neutrophil-activating peptide 78 (ENA-78)
Interleukin-10	CXCL8/interleukin-8
Interleukin-11	CXCL9/monokine induced by gamma interferon (MIG)
Interleukin-12	CXCL10/interferon γ induced protein 10 (IP-10)
Interleukin-13	CXCL11/interferon-inducible T cell alpha chemoattractant (I-TAC)
Interleukin-16	
Interleukin-17	
Interleukin-25	
Interferon-γ (IFNγ)	
Tumor necrosis factor-α (TNF)	

## Known mechanism of eosinophils with potential roles in BP (hypothesis)

### Learning from other eosinophil-mediated conditions

Numerous conditions involve a predominance of eosinophils such as allergic reactions, parasitic infections, and certain malignancies. From these, a large amount of information regarding the function of eosinophils has been discovered. However, it is important to note that not all eosinophils are the same, even within the same disease and organ system. Eosinophils differ in their molecular pattern as observed by Lingblom et al. when investigating differences in children and adults with eosinophilic esophagitis (123). Moreover, investigations in healthy individuals revealed age-dependent differences in levels of eosinophil markers. For instance, levels of CD44 increased with age, while levels of CD54, prostaglandin DP2 receptor (CRTH2), and galectin-10 decreased with age. In addition, they demonstrated that young healthy children express highest levels of galectin-10, CRTH2, and CD54 and that these diminish with age (123). Similarly, the gastrointestinal system hosts substantial number of eosinophils exhibiting differences to eosinophils in the lungs or blood. For example, intestinal eosinophils rarely degranulate, and their lifespan is far longer than of those found in inflammatory sites (124, 125).

Despite these limitations, many functions of eosinophils appear to be retained across allergic diseases. To what degree they contribute specifically to BP, however, remains to be determined. We thus review these known mechanisms, which have a scientific rationale for contributing to the pathogenesis and symptomatology of BP.

### The role of eosinophils in BP related pruritis

Pruritis is a hallmark of BP. In certain cases, it can be the presenting symptom, even when a rash is not present (16). While the depletion of BP180 in itself can generate itch as seen in BP180 knockout mice (126), several other pathways potentially contribute to this cardinal symptom of BP.

#### Interleukin-31

IL-31 belongs to the IL-6 family of cytokines produced in part by activated Th2 cells (127). It has a significant role in itch, by activating endothelin-1 responsive neurons and by increasing the release of brain natriuretic peptide (BNP), a central mediator of itch (128, 129). IL-31 additionally induces cutaneous nerve growth and branching (130).

In BP, elevated levels of IL-31 have been demonstrated in serum and in lesional skin of patients (131) and has been significantly associated with both eosinophilia and elevated anti BP-180-IgE (132). Eosinophils are capable of producing IL-31 (132, 133). In fact, eosinophils were recently shown to be the primary source of IL-31 in BP (134).

#### Substance P

Substance P is a major pruritogen and vasodilator released from peripheral nerves. The presence of substance P in BP has, however, varied between studies (135, 136). Substance P can have significant interactions with eosinophils. In mouse models of atopic dermatitis, degranulated eosinophils have been found surrounding an increased number of substance P-positive nerve fibers in lesional skin (137). Also, nasal provocation with substance P in patients with allergic rhinitis leads to an increased number of eosinophils (138, 139). Substance P acts on cells via binding to neurokinin-1 receptor (NK1R) and neurokinin-2 receptor (NK2R). Interestingly, effects of substance P on eosinophils include inhibition of apoptosis in a comparable manner to IL-3, a known apoptosis inhibitor, which contribute to extend eosinophil survival and may perpetuate its biological effects in disease (139).

Substance P can also induce the release of nerve growth factor (NGF) and IL-31 from eosinophils, in addition to mast cells. NGF may play a significant role mediating pruritus due to its ability to sensitize primary itching sensing neurons (140). NGF released from eosinophils may then stimulate neighbor nerves to further release substance P. Other roles of substance P on eosinophils include chemotaxis, activation and survival, thus potentially perpetuating the itch cycle. Mast cell-eosinophil crosstalk can also develop a neuro-immune communication axis and subsequently induce distinctive substance P itch (140).

#### Direct interaction with peripheral nerves

Eosinophils interact with nerve cells leading to enhanced growth and branching resulting in enhanced innervation of the skin, as documented in cultured dorsal root ganglion neurons (141, 142). Eosinophils also coordinate changes in neurotransmitter release, and protection from cytokine-induced apoptosis. In part, these interactions occur as a result of activation of neural NFκB, activated by adhesion of eosinophils to neural intercellular adhesion molecule-1 (ICAM-1) (143).

The close relationship of eosinophils and nerves has been demonstrated in human skin samples of atopic dermatitis patients where investigators observed increased nerve density near eosinophil granule proteins. These findings were reproduced in mice whereby histological samples of murine skin showed that IL-5-stimulated eosinophils were present in the same epidermal foci of increased nerves. *In vitro* experiments with cultures of eosinophils have shown a dramatic increase in branching of sensory neurons. Collectively, these findings are in favor of an important role for eosinophils in cutaneous nerve growth (141).

Eosinophil granules may mediate a crosstalk between nerves and eosinophils. In histological samples of prurigo nodularis patients, for example, ECP- and EDN/EPX-containing eosinophils were primarily distributed in the upper dermis where nerves were also increased in number. Some of these nerves were even in direct contact with eosinophils (144).

#### Interaction with the autonomic nervous system

Eosinophils and the autonomic nerve system demonstrate a two-way cross talk. *In vitro* experiments demonstrate that the adherence of eosinophils to cholinergic nerves triggers a series of molecular events including activation of NFκB and activator protein (AP)-1 in the nerve cells, ultimately promoting nerve growth (145). In guinea pigs, adhesion of eosinophils to parasympathetic nerves results in release of reactive oxygen species (ROS) via neuronal NADPH oxidase, as well as activation of p38 MAP kinase (146). Eosinophils have also been implicated in the remodeling of neurites of the cholinergic nerve cell line (146).

Individual eosinophil derived granule proteins have been shown to affect cholinergic nerves. At non-cytotoxic concentrations, eosinophil cationic proteins have been shown to induce nerve cell signaling pathways by phosphorylation of the MAP kinases ERK 1/2, p38, and AKT and subsequent activation of the nuclear transcription factor NFκB (147). EPX has been shown to upregulate choline acetyltransferase (ChAT) and vesicular acetylcholine transporter (VAChT) gene expression while MBP upregulated VAChT alone. These enzymes coordinate the production of acetylcholine whereby ChAT catalyzes the production of acetylcholine from choline and acetyl-CoA, and VAChT regulates packaging into vesicles for synaptic release (148).

MBP and NGF have also been implicated in upregulating muscarinic M2 receptor expression *in vitro;* observed changes were associated with a reduction of intracellular neural acetylcholine and an increase in choline content (149, 150). MBP also protects nerve cells from apoptosis by upregulation of adhesion-dependent activation of ERK1/2, inducing expression of the antiapoptotic gene bfl-1 and bfl-2 (147, 149, 151). Thus, MBP released from eosinophils at inflammatory sites may regulate peripheral nerve plasticity by inhibiting apoptosis (147). In animal models, eosinophil MBP is associated with the hyperreactivity of cholinergic nerves (148, 152–155).

Cholingeric nerves additionally can influence eosinophils. Eosinophil degranulation has been demonstrated in tissue taken from patients with inflammatory bowel disease and asthma in which eosinophils adhered to cholinergic nerves (156, 157). Interestingly, nicotinic agonists decrease eosinophil infiltration in lungs and airways of mice (158).

### Eosinophils act as antigen presenting cells

While eosinophils had traditionally been considered an effector cell, recent advances have elucidated the multifaceted nature of eosinophils which can affect tissue homeostasis, metabolism, and immune regulation in both disease and the steady state (159). Eosinophils can effectively process antigen, express co-stimulatory molecules, traffic to the lymph node and induce a T-cell response (160–163). This can be stimulated by GM-CSF. Studies in wild-type mice have demonstrated that eosinophils in the lamina propria of the intestine express surface markers such as MHC II and CD80 suggesting that eosinophils in this location are capable of functioning as APCs. Moreover, investigators identified intraepithelial eosinophils exhibiting dendrites with extensive reaches. Further experiments with antigen sensitized mice revealed that despite the presence of two distinct populations, both populations of eosinophils acquired intestinal antigen *in vivo* (159). Double stain-immunohistochemistry has likewise been used to demonstrate T-cell activation and tissue eosinophils expressing MHC-II in specimens of eosinophilic esophagitis (164). Still, the efficiency of eosinophils as APCs, their function in the skin, and their ability to process key antigens in BP remains unknown.

### Direct pathogenic actions of degranulation proteins and reactive oxygen species

Eosinophil degranulation proteins are capable of inducing cytotoxicity through several mechanisms. ECP induces pore formation in the cell membranes contributing to inflict cellular damage (58), while MBP increases smooth muscle reactivity due to selective allosteric antagonism of vagal muscarinic M2 receptors (165) and triggers degranulation of mast cells and basophils (166, 167). While we demonstrated a cytotoxic effect of degranulation proteins on keratinocytes at physiologic doses seen in BP (96), it is unclear the extent of damage *in vivo* as necrosis is not a histologic feature of BP.

Eosinophil peroxidase generates hydrogen peroxidase as well as superoxide, causing additional damage (168). Interestingly, blockade of ROS was capable of inhibiting blister formation in an *ex vivo* model of BP (169). Limitations to the cryosection model however may overstate the role of ROS in DEJ separation. A similar study in neutrophil mediated BP evaluated luteolin, a plant-derived flavonoid with potent anti-oxidative and anti-inflammatory properties effects in *ex vivo* cryosection model of BP, resulting in a significant reduction of autoantibody-induced DEJ separation. However *in vivo* mouse experiments did not yield comparable results (170). Thus, further *in vivo* studies are needed to determine the role of antioxidants in inhibiting eosinophil induced ROS in BP.

### Can eosinophils sustain local immune response?

Eosinophils express a series of cytokines involved in plasma cell survival such as activation and proliferation-induced ligand (APRIL), IL-6, IL-4, IL-5, IL-13, IL-10, and TNF (171, 172). Therefore, they could in theory provide a local stimulus sustaining Ig producing plasma cells in the dermis. Eosinophil IL-16 a key cytokine in T-cell recruitment, as well as IL-4, IL-5, and IL-13 can stimulate Th2 immunotype. Thus, eosinophils could also in theory perpetuate T-cell stimulation and a Th2 milieu.

#### Effect of eosinophils on B-cells

B-cell responses are regulated by a series of signals including IL-2, IL-4, IL-7, IL-15, and members of the TNF family such as CD40 ligand (173). In the past two decades, two TNF family molecules: B cell-activating factor of the TNF family (BAFF) and APRIL have been recognized as key regulators of normal B cell functions and autoimmune B cell induction, both of which are expressed by eosinophils (174).

APRIL binds to receptors such as transmembrane activator and calcium modulator ligand interactor (TACI) and B cell maturation antigen (BCMA) (175, 176). Normal functions of APRIL include: increasing B-cell antigen presentation, stimulation of antigen-activated B-cells, enabling isotype switching in B cells, and augmenting plasma cell survival (174, 177, 178). BAFF acts as a potent B-cell growth factor as well as stimulus for immunoglobulin production (173, 179).

BAFF levels are significantly elevated in patients with BP. Interestingly, BAFF levels increased before the anti-BP180 antibody level and quickly decreased in response to treatment, making it a useful marker for early disease (180). Levels of APRIL are likewise elevated in BP, and closely correlate with BAFF. Levels of APRIL similarly occur extremely early on in the development of disease, and thus appear to be a key mediator prior to the development of detectable levels of autoantibodies (181). Whether the APRIL and BAFF in BP primarily comes from eosinophils or other immune cells remains unknown.

#### Effect of eosinophils on T-cell recruitment

CCL5 (RANTES) is a chemoattractant for CD4^+^ memory T cells, monocytes, and eosinophils (182, 183). *In vitro* studies have shown that RANTES activates T lymphocytes in an antigen-independent manner (184). RANTES may also activate eosinophils, upregulating their expression of adhesion molecules and enhance transendothelial migration (182, 183, 185, 186). IL-16 is likewise a strong attractant of CD4+ T-cells. Eosinophils release both IL-16 and RANTES. Even at very low concentrations, RANTES and IL-16 induce migration of T-lymphocytes. Thus, eosinophils can potentially amplify the immune response by recruitment of CD4+ lymphocytes as well as additional eosinophils (187). These interactions have yet to be elucidated in BP.

#### Effect of eosinophils on Th2 polarization

Th2 polarization may be driven by eosinophil production of IL-4, IL-5, and IL-13 (188). In addition, eosinophil expression of indoleamine 2,3-dioxygenase (IDO) which catalyzes the conversion of tryptophan to kynurenines, can regulate T cell subset selection toward Th2 (189). Thus, eosinophil products can serve as Th2 adjuvants via dendritic cell regulation (190, 191).

Eotaxin and MCP-4 are two chemokines that play a significant role in the selective recruitment of not only Th2 effector cells, but also eosinophils to the inflammatory site of BP, both of which are present at elevated levels in tissue and blister fluid (79, 122, 192). In a series of experiments investigators have found that eotaxin and MCP-4 mRNA were expressed in all biopsies of BP patients, present in the epidermis, and were also expressed in eosinophils. Immunohistochemical studies confirmed that these chemokines were localized to the granules of eosinophils (79). Overall, the levels of Th2 associated chemokines (eotaxin and MCP-4) in blister fluid are significantly greater than Th1 associated chemokines (MIP-1B and IP-10). Whether eosinophils or keratinocytes are the primary source of these chemokines is unclear (193).

### Keratinocytes exposed to anti-BP180 antibodies express key cytokines and chemokines involved in eosinophil chemotaxis

In BP, eosinophils are classically aligned along the basement membrane, with several eosinophils often traveling into the epidermis via exocytosis. While eosinophil binding to the BMZ is known to require IgG and complement (not IgE), the ability of eosinophils to exocytose into the epidermis likewise cannot sufficiently be explained by this. Thus, keratinocyte signaling is likely to have a pivotal role, as keratinocytes can express key chemokines such as IL-8 and eotaxins.

#### Interleukin-8

IL-8 is produced by keratinocytes when exposed to BP autoantibodies (42, 44, 194–197). IL-8 is a known chemoattractant for neutrophils (198, 199). Studies investigating relationship of neutrophils, eosinophils and IL-8 have shown that IL-8 stimulates neutrophils to induce trans-basement membrane migration of eosinophils in the airways of asthmatic patients (200). Upon lipopolysaccharide (LPS) and IL-8 stimulation, neutrophils produce several chemoattractants for eosinophils such as leukotriene B4 and PAF that can recruit eosinophils and induce trans-BMZ migration (201, 202). Interestingly, IL-8 does not stimulate eosinophils alone to migrate through artificial BMZ (Matrigel) (203). Therefore IL-8 seems to play a key role to stimulate neutrophils resulting in subsequent trans-basement membrane migration of eosinophils (197).

Autoantibodies to BP180 mediate the release of IL-8 from human keratinocytes in a dose and time dependent manner (42, 195). In fact, this cytokine is known to be elevated in sera and blister of BP patients with significantly higher levels of IL-8 in blister fluid as compared to serum (42, 194, 196).

#### Eotaxins

Aside from stimulating Th2 polarization, eotaxins have a key role in the attraction of eosinophils into their target tissues. Eotaxins consists of three chemokines CCL11, CCL24, and CCL26 (204). The main eotaxin receptor is CCR3 which is expressed on all eosinophils in peripheral circulation (205).

The presence of elevated levels of eotaxin, IL-5 and CCR3 has been demonstrated in blister fluid, lesional and perilesional skin in BP (77, 122). Significant correlation with these markers and the number of dermal infiltrating eosinophils has also been demonstrated (77). In addition, studies investigating specific ligands have shown that CCL11 and CCL26 are significantly associated with activated eosinophils (204). Epidermal expression of eotaxin appears to be a consistent feature among all eosinophilic dermatoses (193).

## Conclusion

Eosinophils are complex cells with numerous functions. They generally make up the predominant inflammatory cell-type seen in BP. In recent years, the overall understanding of eosinophils has significantly improved, leading to new avenues to pursue in the pathogenesis of BP. Several eosinophil pathways have well-defined roles in the pathogenesis of BP that demonstrate not only a correlative role in disease and severity, but rather a causative.

- MMP9 is secreted from eosinophils and is capable of cleaving BP180 and activating NE
- Eosinophil degranulation proteins are deposited on basal keratinocytes
- Eosinophil extracellular traps can contribute to DEJ separation. This can be abrogated with DNAse treatment
- BP180 IgE autoantibodies need eosinophils in order to mediate DEJ separation *in vivo*. This requires FcεRI which while not typically expressed on eosinophils, is significantly overexpressed in BP.
- Eotaxin and MCP-4 are seen in eosinophil granules in BP patients, thus perpetuating tissue eosinophilia.

Aside from these known direct roles in the pathogenesis of BP, several known functions of eosinophils have a scientific rationale to contribute to symptomatology and the pathogenesis of BP. Limitations to drawing further conclusions are summarized below:

- Eosinophils are the key producer of IL-31, a known pruritogen, in BP. Whether this is the primary pruritogen in BP is not known.
- Eosinophils can directly attach or degranulate onto peripheral and autonomic nerves, inducing branching and nerve growth which can lead to pruritis. While known to occur in other skin disease, this has not been studied in BP.
- Eosinophils can act as functional APCs. It is not known whether eosinophils in the skin can function as APCs, and whether they can effectively process the BP180 antigen
- Eosinophil degranulation proteins are known to be cytotoxic and have been shown to be cytotoxic to keratinocytes. Whether this cytotoxicity has *in vivo* contributions to the pathogenesis of BP has not been studied.
- Eosinophils cause generation of ROS which when blocked in *ex vivo* models, can prevent DEJ separation. Whether eosinophil induced ROS is sufficient to lead to disease *in vivo* has not been studied. In neutrophils, this is not sufficient to prevent blister formation.
- Eosinophils are known to produce BAFF and APRIL, two key regulators of autoimmune B-cells. Whether they produce BAFF and APRIL in BP, and whether this is indispensable in promoting B-cell responses in BP is not known.
- Eosinophils are known to secrete IL-16 and RANTES, two key T-cell recruiting molecules. Whether this occurs in BP, and whether this is indispensable for T-cell involvement in BP is not known.
- Eosinophils are known to secrete IL-4, IL-5, and IL-13 which can promote Th2 polarization. Whether they are the primary source of IL-4, IL-5, and IL-13 in BP, as well as whether they are indispensable in promoting Th2 polarization in BP is not known.
- Keratinocytes express Eotaxin and IL-8, both strong attractants for eosinophil migration. Whether this has pathologic significance is not known.

**Figure 2:**
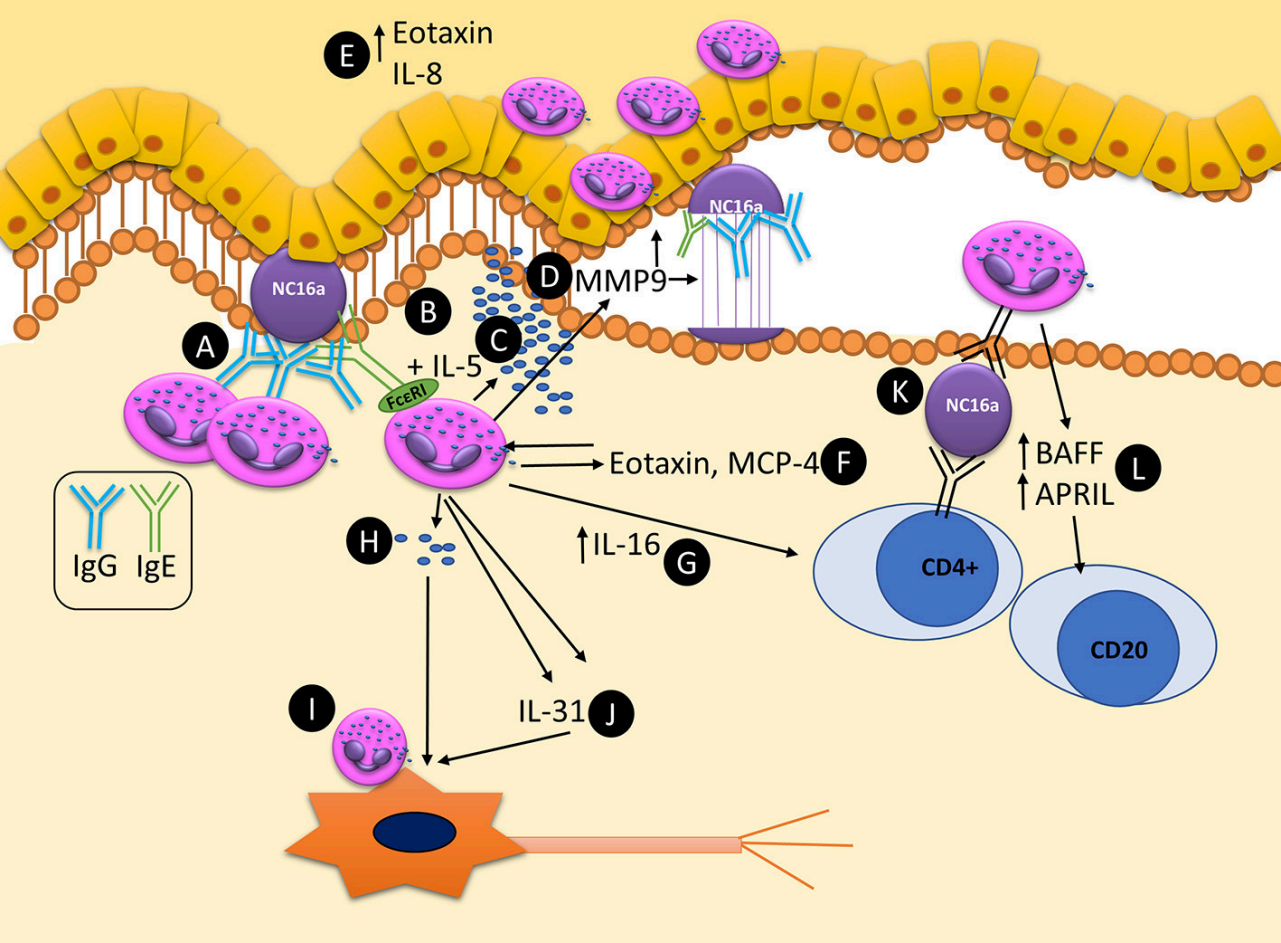
Schematic representation of known and potential pathways by which eosinophils can contribute to the pathogenesis and maintenance of autoimmunity in bullous pemphigoid. **(A)** Eosinophils bind anti-BP180 IgG, aligning along the BMZ. **(B)** Dermal eosinophils express FcεRI which can bind to anti-BP180 IgE leading to DEJ separation. **(C)** Upon activation with IL-5 eosinophils can lead to DEJ separation and degranulation. **(D)** MMP9 is secreted from eosinophils and is capable of cleaving BP180. **(E)** Eotaxin and IL-8 are expressed in the epidermis, acting as eosinophil chemotactic chemokines, attracting further tissue eosinophilia. **(F)** Eotaxin and MCP-4 are released from eosinophil granules, further driving tissue eosinophilia and Th2 polarization. **(G)** IL-16 is released from eosinophils and is capable of stimulating T-cell response. **(H)** Eosinophils can directly degranulate on and **(I)** directly bind to neurons leading to increase branching and potentially pruritis. **(J)** Eosinophils secrete IL-31, a major pruritogen which can stimulate nerves. **(K)** Eosinophils are capable of acting as antigen presenting cells, potentially leading to T-cell responses by binding bound antigen via MHC-II to T-cell receptors. **(L)** Eosinophils express BAFF and APRIL, potentially stimulating local autoimmune B-cells.

The principle mechanism by which eosinophils can potentially contribute to the pathogenesis of BP are summarized as a schematic in Figure 2. Future studies addressing these uncertainties will provide a more thorough understanding of the roles of eosinophils in BP, as well as eosinophils in the skin.

## Author contributions

KA: Substantial contributions to the conception or design of the work, or the acquisition, analysis, or interpretation of data for the work; KA, MV, KK, SG: Drafting the work or revising it critically for important intellectual content, Final approval of the version to be published, Agreement to be accountable for all aspects of the work in ensuring that questions related to the accuracy or integrity of any part of the work are appropriately investigated and resolved.

### Conflict of interest statement

The authors declare that the research was conducted in the absence of any commercial or financial relationships that could be construed as a potential conflict of interest.

## Abbreviations

AP-1: activator protein-1
APC: antigen presenting cell
APRIL: activation and proliferation-induced ligand
BAFF: B cell-activating factor
BMZ: basement membrane zone
BNP: brain natriuretic peptide
BP: bullous pemphigoid
C3: Complement 3
CCL5: RANTES (regulated on activation, normal T-cell expressed and secreted)
CCL11: Eotaxin-1
CCL24: Eotaxin 2
CCL26: Eotaxin 3
ChAT: choline acetyltransferase
CRTH2: prostaglandin DP2 receptor
DEJ: Dermal-epidermal junction
ECP: eosinophil cationic protein
EDN: eosinophil derived neurotoxin
EET: eosinophil extracellular traps
EPX: eosinophil peroxidase
FcεRI: human high-affinity IgE receptor
GM-CSF: granulocyte-macrophage colony-stimulating factor
IIF: indirect immunofluorescence
ICAM-1: intercellular adhesion molecule-1
IP-10: IFN- γ- inducible protein 10
MBP: major basic protein
MCP: monocyte chemoattractant protein
MIG: monokine induced by IFN- γ
MMP-9: Metalloprotease 9, Gelatinase B
NC16a: non-collagenous 16A domain of BP180
NE: neutrophil elastase
NGF: nerve growth factor
NK1R: neurokinin-1 receptor
NK2R: neurokinin-2 receptor
PAF: platelet activating factor
ROS: reactive oxygen species
TNF-α: tumor necrosis factor-α
VAChT: vesicular acetylcholine transporter
VCAM-1: vascular cell adhesion molecule-1.
